# Supplementary material for: A Staged Approach using Machine Learning and Uncertainty Quantification to Predict the Risk of Hip Fracture
Source: ArXiv. 2024 May 30:arXiv:2405.20071v1. Preprint. [Version 1] (PMC11160872)
Supplement: Supplement 1 [file NIHPP2405.20071v1-supplement-1.pdf]

Supplementary Materials

Table 1. List of clinical variables used in this study.

Feature	Meaning	Range
**Age**	Age of the participant on the day of attending an Initial Assessment Centre, truncated to whole year.	Integer, years
**Sex**	Gender of the participant.	Categorical<br>Female (0), Male (1)
**Genetic sex**	Biological sex of the participant.	Categorical<br>Female (0), Male (1)
**Weight**	Weight measured during the initial assessment center visit and amalgamated into a single item.	Continuous, Kg
**Average total household income before tax**	Total household income before tax received by the household, categorized into income brackets.	Categorical<br>(Various income ranges)
**Smoking status**	Current or past smoking status of the participant.	Categorical<br>(Never, Previous, Current)
**Alcohol drinker status**	Current or past alcohol consumption status of the participant.	Categorical<br>(Never, Previous, Current)
**Variation in diet**	Frequency of diet variation.	Categorical<br>(Never/rarely, Sometimes, Often)
**Major dietary changes in the last 5 years**	Major changes made to the diet in the last 5 years.	Categorical<br>(No, Yes because of illness, Yes because of other reasons)
**Falls in the last year**	Frequency of falls in the last year.	Categorical<br>(No falls, Only one fall, More than one fall)
**Fractured/broken bones in last 5 years**	History of fractured or broken bones in the last 5 years.	Categorical: Yes, No, Do not know, Prefer not to answer
**Femur neck BMD (right)**	Bone mineral density of the right femur neck.	Continuous, g/cm2
**Femur neck BMC (right)**	Bone mineral content of the right femur neck.	Continuous, g
**Femur total BMD (right)**	Bone mineral density of the right femur.	Continuous, g/cm2
**Femur total BMD T-score (right)**	T-score of the right femur total bone mineral density.	Continuous, Std.Devs
**Femur troch BMD (right)**	Bone mineral density of the right femur trochanter.	Continuous, g/cm2
**Femur troch BMD T-score (right)**	T-score of the right femur trochanter bone mineral density.	Continuous, Std.Devs
**Femur wards BMD (right)**	Bone mineral density of the right femur Ward's triangle.	Continuous, g/cm2
**Femur wards BMD T-score (right)**	T-score of the right femur Ward's triangle bone mineral density.	Continuous, Std.Devs
**Femur neck BMD (left)**	Bone mineral density of the left femur neck.	Continuous, g/cm2
**Femur neck BMC (left)**	Bone mineral content of the left femur neck.	Continuous, g
**Femur total BMD (left)**	Bone mineral density of the left femur.	Continuous, g/cm2

**Femur total BMD T-score (left)**	T-score of the left femur total bone mineral density.	Continuous, Std.Devs
**Femur troch BMD (left)**	Bone mineral density of the left femur trochanter.	Continuous, g/cm2
**Femur troch BMD T-score (left)**	T-score of the left femur trochanter bone mineral density.	Continuous, Std.Devs
**Femur wards BMD (left)**	Bone mineral density of the left femur Ward's triangle.	Continuous, g/cm2
**Femur wards BMD T-score (left)**	T-score of the left femur Ward's triangle bone mineral density.	Continuous, Std.Devs
**Pelvis BMC**	Bone mineral content of the pelvis.	Continuous, g
**Vitamin and mineral supplements**	Regular intake of various vitamins and minerals.	Categorical
